# Developing LRP1 Agonists into a Therapeutic Strategy in Acute Myocardial Infarction

**DOI:** 10.3390/ijms20030544

**Published:** 2019-01-28

**Authors:** Nicola Potere, Marco Giuseppe Del Buono, Giampaolo Niccoli, Filippo Crea, Stefano Toldo, Antonio Abbate

**Affiliations:** 1 VCU Pauley Heart Center, Virginia Commonwealth University, Richmond, VA 23298, USA; nic.potere@gmail.com (N.P.); marcodelbuono@hotmail.it (M.G.D.B.); stefano.toldo@vcuhealth.org (S.T.); 2 Unit of Cardiovascular Sciences, Department of Medicine, Campus Bio-Medico University of Rome, 00128 Rome, Italy; 3 Department of Cardiovascular and Thoracic Sciences, Fondazione Policlinico Universitario A. Gemelli, IRCCS, Università Cattolica del Sacro Cuore, 00168 Rome, Italy; gniccoli73@hotmail.it (G.N.); filippo.crea@unicatt.it (F.C.)

**Keywords:** cardioprotection, ischemia-reperfusion injury, RISK pathway, LRP1

## Abstract

Cardioprotection refers to a strategy aimed at enhancing survival pathways in the injured yet salvageable myocardium following ischemia-reperfusion. Low-density lipoprotein receptor-related protein 1 (LRP1) is a multifunctional receptor that can be targeted following reperfusion, to induce a cardioprotective signaling through the activation of the reperfusion injury salvage kinase (RISK) pathway. The data from preclinical studies with non-selective and selective LRP1 agonists are promising, showing a large therapeutic window for intervention to reduce infarct size after ischemia-reperfusion. A pilot clinical trial with plasma derived *α*1-antitrypsin (AAT), a naturally occurring LRP1 agonist, supports the translational value of LRP1 as a novel therapeutic target for cardioprotection. A phase I study with a selective LRP1 agonist has been completed showing no toxicity. These findings may open the way to early phase clinical studies with pharmacologic LRP1 activation in patients with acute myocardial infarction (AMI).

## 1. Introduction

Acute myocardial infarction (AMI) is a leading cause of morbidity and mortality world-wide [1]. The primary cause of AMI is atherothrombosis of a coronary artery plaque following an abrupt plaque destabilizitation [1]. Obstruction of the flow by atherothrombosis leads to acute myocardial ischemia and subsequent cardiomyocyte necrosis [1]. Prompt reperfusion, using either antithrombotic therapies and/or percutaneous coronary intervention, is the most effective strategy to reduce myocardial ischemic injury and improve clinical outcomes in patients with AMI [2]. Despite the timely and effective current myocardial reperfusion strategies, and the ensuing reduction in infarct size, reperfusion is unable to completely halt the progression of the injury wave [3,4]. Ischemia-reperfusion injury (IRI) refers to the phenomenon by which the injured yet still viable myocardium, which is in a delicate death–survival balance, is injured by the re-establishment of blood flow and the re-oxygenation of the ischemic myocardium, which is therefore only incompletely salvaged by reperfusion [4]. This secondary wave of injury that occurs despite reperfusion, is a significant contributor to the size of the infarct [4,5,6]. Hence, experimental strategies aimed at limiting IRI in AMI are expected to reduce the infarct size by at least an additional 25%, aiding in the prevention of left ventricular adverse remodeling and heart failure [4,5,6,7,8].

Programmed cell death plays a central role in determining the fate of the injured-yet-salvageable myocardium [9,10,11]. Concomitant and competing signaling are activated in the myocardium after reperfusion, promoting cell death on the one hand, and cell survival on the other [10]. The stimuli that regulate one versus the opposing pathways are incompletely characterized.

We herein describe the potential role of low-density lipoprotein receptor-related protein 1 (LRP1) signaling, and its modulation in the regulation of cell survival pathways and reduction of IRI.

## 2. Pathophysiology of Ischemia-Reperfusion Injury (IRI)

Ischemic necrosis is preventable and/or minimized by reperfusion. However, reperfusion itself paradoxically accelerates a series of events that result in cellular injury and death [3,4,5]. Several mechanisms are implicated in the pathogenesis of IRI, including abnormalities in energy production and utilization, alterations of sub-cellular architecture, and different forms of regulated cell death (i.e., necrosis, apoptosis, and pyroptosis) [5,9,12,13].

Reperfusion may exacerbate the damage induced by ischemia through further enhancement of necrotic cell death [14]. Necrosis, initially considered an accidental and unregulated modality of cell death, has recently emerged as a form programmed cell death [14,15]. The mitochondria have indeed emerged as important regulators of both apoptotic and regulated necrotic cell death. The extensive production of reactive oxygen species (ROS), the high intramitochondrial Ca^2+^ phosphate levels, and adenosine nucleotide depletion create the optimal conditions for a prolonged mitochondrial permeability transition pore (mPTP) opening through the matrix protein cyclophilin D (CypD), leading to an energetic collapse and irreversible cell damage and death [14,15].

Apoptosis is a finely regulated form of programmed, non-inflammatory, cell death, triggered by reperfusion and due to the rapid restoration of the mitochondrial activity, pH imbalance, and adenine nucleotides synthesis and release, following a period of prolonged ischemia [9]. The opening of mPTP and mitochondrial swelling and rupture also lead to the release of pro-apoptogenic mediators, such as cytochrome c, second mitochondria-derived activator of caspase/direct inhibitor of apoptosis-binding protein with low pI (Smac/DIABLO), Omi/HtrA2, apoptosis-inducing factor (AIF), and endonuclease G (EndoG), from the mitochondrial intermembrane space, promoting caspase-3 activation and resulting in cardiomyocyte apoptosis [9]. Differently from the necrotic process, caspase activation is an ATP-dependent process requiring some degree of mitochondrial function [15].

Pyroptosis is a form of inflammatory programmed cell death, morphologically and functionally different from apoptosis, and mediated by caspase-1 [6]. Caspase-1 is not part of the canonical apoptotic pathway, and it is activated during pyroptosis by a large supramolecular protein complex, termed inflammasome, which has been widely described as the culprit for the production and secretion of the powerful pro-inflammatory cytokines interleukin-1β (IL-1β) and interleukin-18 (IL-18) [16]. The most widely characterized inflammasome sensors in the heart are NACHT, LRR, and PYD domains-containing protein 3 (NLRP3), which, during AMI, are activated in response to the cellular debris released by injured tissue in the form of intracellular and extracellular, structurally different molecules, collectively known as damage associated molecular patterns (DAMPs) [6,8]. NLRP3 inflammasome expression and activity are enhanced after one to three hours after reperfusion [8,17]. However, NLRP3 also displays signaling functions independent from the inflammasome, which may also have a role in IRI [18].

Numerous studies have been performed to determine the nature of the key death and survival pathways following myocardial ischemia and reperfusion, and several attempts to improve the outcome of reperfusion strategies have followed [5,19]. Identifying the key pathophysiologic mechanisms of IRI may indeed lead to a smaller infarct size and reduced early and long-term mortality in patients with AMI [4,5]. Despite the numerous efforts in understanding the pathophysiology of this process and the encouraging preclinical studies with promising agents [5,19], there are still no cardioprotective strategies approved to limit IRI in clinical practice [7]. The delicate balance between death and survival pathways has been shown to be extremely time-sensitive, limiting the opportunities to intervene [19]. However, because of the complexity of the pathophysiology of IRI, targeting exclusively one of the mechanisms implicated in IRI (i.e., necrosis, apoptosis, or pyroptosis) may not be sufficient to completely prevent IRI.

## 3. The Reperfusion Injury Salvage Kinase (RISK) Pathway

The RISK pathway refers to a complex signaling cascade that combines two main pro-survival pathways, the phosphatidylinositol-4,5-bisphosphate 3-kinase (PI3K) and PI3K-protein kinase B (Akt), together with the mitogen-activated protein kinase kinase (MEK) and extracellular signal-regulated kinase 1/2 (ERK1/2) [10,20,21].

The evidence supporting the key role for the RISK pathway derives from the cardioprotective effects observed with pharmacological agents such as urocortin, which are abolished by the co-administration of PI3K-Akt and MEK-ERK1/2 inhibitors [21]. Several pharmacological (i.e., insulin-like growth factor-1, transforming growth factor-β1, cardiotrophin-1, atorvastatin, and bradykinin) and non-pharmacological (i.e., ischemic pre- and post-conditioning) interventions were similarly shown to stimulate the RISK pathway, to confer cardioprotection upon reperfusion [22]. Therefore, the RISK pathway is nowadays considered the prevailing signaling cascade in cardioprotection following IRI [22].

Recent studies have explored the molecular mechanisms downstream of PI3K-Akt and the ERK1/2, and their interplay to mediate cardioprotection at reperfusion [23]. In brief, the activation of the Akt and/or ERK1/2 kinase cascades results in (1) the indirect inhibition of apoptosis, by the deactivation of key pro-apoptotic proteins thus preventing cytochrome c release from the mitochondria [24,25,26]; (2) the inhibition of mPTP opening (leading to abolition of mitochondrial functions and myocyte necrosis) and the activation of the endothelial nitric oxide synthase (eNOS) [27,28]; (3) the phosphorylation of factors involved in the regulation of gene expression [29,30,31]; (4) and the direct inhibition of apoptosis, by inactivating caspase-3 and -9 [32,33].

The PI3K-Akt signal transduction cascade is mediated by class IA PI3K. PI3K p110α is coupled to the p101 adaptor protein that is conventionally activated by growth factors through G-protein-coupled receptors (GPCR), participating in the regulation of cell survival, growth, and metabolism [10]. There are several isoforms of PI3K that differ in their preferential expression in different cell types; different locations within the cellular compartments; pairing with different receptors; and phosphorylating a diverse array of substrates, including glycogen synthase kinase-3 (glycogen and protein metabolism), apoptotic proteins (Bad, Bax, Bim, p53 and caspase), GLUT4 vescicles (glucose metabolism), transcription factors (IKK-α and Forkhead proteins), p70S6K, eNOS, and protein kinase c (PKC) [10]. The PI3K produces phosphatidylinositol (3,4,5)-trisphosphate (PIP3), which recruits cytosolic Akt to the plasma membrane, which is then phosphorylated on Thr(308) by the pyruvate dehydrogenase kinase 1 (PDK1) and on Ser(473) by the mammalian target of rapamycin (mTORC) [10]. Upon full activation, Akt interacts with over 70 known downstream molecular targets [10].

Similarly, the MEK-ERK1/2 pathway (also known as p42/44 MAP kinase pathway) is conventionally activated by tyrosine kinase receptors, G-protein-coupled receptors, and environmental stimuli, and controls cell proliferation, differentiation, and survival [34,35,36]. The cascade involves the activation of the MEK1/2 by c-Raf, which activates ERK1/2, which, in turn, directly phosphorylates over 160 targets [34,35,36].

Both Akt and ERK promote mTORC1 signaling, a crucial pathway in cell growth, proliferation, and metabolism, through phosphorylation on the distinct sites of a repressor of mTORC1 activity, a GTPase activator protein, named tuberous sclerosis complex 2 (TSC2) [37]. These events lead to a greater repression of its activity, resulting in a synergic and more powerful stimulation of mTORC1 signaling [37].

The endogenous activation of the RISK pathway during the early reperfusion phase following ischemia limits reperfusion injury. Therefore, the pharmacological stimulation of the RISK pathway represents a therapeutic strategy to reduce reperfusion-mediated cardiomyocyte death. The activation of the PI3K and ERK1/2 kinase cascades appears to constitute a universal pro-survival kinase cascade that mediates cardioprotection at reperfusion [10,22].

Necrosis, apoptosis, and pyroptosis are distinct forms of regulated cell death that, however, are not completely independent from each other [15]. Outer mitochondrial membrane rupture following mPTP opening leads to the cytoplasmic release of proteins, such as cytochrome c, from the intermembrane space, activating the apoptotic pathways [9,15]. The activation of the inflammasome, along with subsequent caspase-1 and pyroptosis, leads to the release of the cardio-depressant cytokines IL-1β and IL-18 in the paracrine milieu, and regulates cell death [6]. Furthermore, in some instances, it has been shown that a key inflammasome component, the apoptosis-associated speck-like protein containing a caspase activation and recruitment domain (CARD) (ASC), which is necessary to activate caspase-1 downstream of NLRP3 activation, can engage with caspase-8, thus powering the molecular cross-talk between apoptosis and pyroptosis [6,38]. Additionally, the pharmacological inhibition of the NLRP3 inflammasome complex has been reported to be associated with the activation of the RISK pathway (i.e., ERK/Akt/GSK-3β signaling) and with the increase in the expression of mitochondrial oxidative phosphorylation markers [17]. This effect may significantly contribute to the cardioprotective effects associated with NLRP3 inhibition, suggesting a converging role of the NLRP3 inflammasome in the modulation of the apoptotic process [16]. In addition, mitochondrial ROS and oxidized adenosine nucleotide generation, promoted by the opening of mPTP, have been suggested to act as a mitochondrial danger signal, promoting the activation of NLRP3 inflammasome [39]. Therefore, NLRP3 inhibition has emerged as a therapeutic strategy to further reduce the infarct size by limiting, together with pyroptosis and necrosis, the deleterious consequences of apoptosis in the setting of IRI.

Nowadays, several approaches have been described to increase the activity of the component of the RISK pathway and to reduce the cell death. For the purpose of this review, we will focus on a potential novel modulator of the IRI, LRP1 [40].

## 4. The Low-Density Lipoprotein (Ldl)-Receptor Related-Protein 1

LRP1, also known as *α*2-macroglobulin receptor (A2MR) or cluster of differentiation 91 (CD91), is an ubiquitous multifunctional membrane receptor of the LDL receptor family, mostly known for its role in lipoprotein scavenging [40,41,42,43,44,45]. Once primarily considered for its role in receptor-mediated endocytosis, it has become clear that LRP1 takes part in multiple signaling pathways involved in protease degradation, glucose homeostasis, modulation of inflammation, coagulation, cell growth, cell survival, and migration [46,47,48,49]. LRP1 has been shown to be involved in several disease processes, such as atherosclerosis, cancer, neurodegenerative, and kidney diseases, as well as, recently, in cardiomyocyte protection during IRI [47,50,51].

Three important properties of LRP1 dictate its diverse role in biology, namely: (1) as a scavenger receptor, LRP1 binds and internalizes a variety of ligands for degradation in lysosomes and recycling through its extracellular domain, such as apolipoprotein E and A2MG; (2) as a signaling receptor, it interacts with numerous adaptor proteins through its cytoplasmic domain in a phosphorylation-specific manner and transduces multiple intracellular signals involved in numerous biological processes, as well as regulates nuclear signaling through the small fragment-LRP1 C-terminal intracellular domain (LRP1-ICD) generated by γ-secretases, and then translocated from the cytoplasm to nucleus; and (3) as a scaffold receptor, it modulates the activity of other membrane proteins such as integrins and receptor tyrosine kinases [40,47,52,53]. These singular properties empower LRP1 to couple to an extracellular microenvironment and an intracellular signaling and response [47].

LRP1 is a type 1 transmembrane protein encoded by the Lrp1 gene located on chromosome 12q13-14 [40]. LRP1 derives from a 600-kDa precursor cleaved by furin-like endoproteases in the trans-Golgi complex to form the mature two-chain structure localized in caveolae, namely: a heavy 515-kDa α-chain coupled through noncovalent interactions to the light 85-kDa β-chain, consisting of a transmembrane segment and a cytoplasmic tail [40,54]. The extracellular α-chain contains four clusters of complement-like repeats, primarily responsible for the ligand-binding activity of LRP1, and epidermal growth factor (EGF) repeats [39]. The light β-chain, which constitutes the transmembrane and the cytosolic domains, includes the tetra amino acidic motif YxxL and dileucine motifs, serving as principal endocytosis signals; two NPxY motifs, functioning as secondary endocytosis signals and binding sites for signaling adapter proteins; and numerous tyrosine residues, whose phosphorylation is essential for LRP1-mediated signal transduction [40,44,45,55,56].

The LRP1 receptor interacts with over 40 distinct molecules, including several plasma proteases [40,45]. Also known as the SERPIN-enzyme complex (SEC) receptor, LRP1 has the ability to recognize the complex of serine proteinase inhibitors (SERPINs) and serine proteases for subsequent endocytosis and degradation [40,57,58]. The binding of the protease-inhibitor complexes to LRP1 is seen across the entire spectrum of SERPINs, such as α1-antitripsin (AAT), A2MG, and antithrombin III (AT_III_). Certain ligands (e.g., SERPINs, tissue-type plasminogen activator -tPA-) function as LRP1 agonists and stimulate LRP1 direct signaling or transactivate signal pathways through LRP1 co-receptors [40,45].

### 4.1. LRP1 and the Modulation of Cell Survival Pathways

Several in vitro and in vivo studies have shown that LRP1 signaling is crucial in inhibiting death pathways and promoting cell survival [51,59,60,61,62,63,64,65,66,67,68,69,70,71,72,73,74,75,76] (Table 1).

LRP1 expression is necessary for the platelet-derived growth factor (PDGF)-mediated activation of the ERK1/2 protein kinase. LRP1 associates with the PDGF receptor-β (PDGFR-β) in the endosomal compartments to modulate its signaling activity on the mitogen-activated protein kinases of the (MAPK)/ERK and PI3K-Akt pathways [59]. The LRP1 cytoplasmic tail is rapidly tyrosine phosphorylated by the PDGF receptor in a src- and PI3K-dependent manner to initiate downstream signaling [60]. Binding of tPA or A2MG to LRP1 in neurons resulted in Src family kinase (SFK) activation and SFK-dependent Trk receptor transactivation, and the subsequent Akt phosphorylation [61]. In neurons, LRP1 induced the phosphorylation of the Ser473 of Akt and reduced apoptosis [62]. The distal NPxY motif in the C terminus of LRP1 was also reported to bridge the N-methyl-D-aspartate (NMDA) receptor through the adaptor protein postsynaptic density protein 95 (PSD95), and initiated tPA-induced ERK1/2 signaling [63,64,65]. Structurally diverse LRP1 agonists, the LRP1-receptor binding domain of A2MG, and hemopexin domain of matrix metalloproteinase 9 (MMP-9), significantly activated and sustained ERK1/2, and enhanced survival in cultured embryonic sensory neurons [66]. In ischemic neurons, the LRP1 antagonism with receptor associated protein (RAP) dramatically reduced the activation of the Akt survival pathway and the levels of the anti-apoptotic factor Bcl-2, while increasing the activation of caspase-3, nuclear-factor kappa B (NF-kB), and MAPKs [67]. Treatment with intravenous immunoglobulin (IVIg), which was previously reported to protect against ischemic stroke-induced brain damage [79,80], reduced these effects in cultured neurons subjected to oxygen-glucose deprivation (OGD) and reperfusion, and significantly increased LRP1 activation in the ischemic hemisphere at 24 h, following middle cerebral artery occlusion (MCAO) and reperfusion [67].

In peripheral nerve injury, LRP1 interacted with various ligands, such as tPA, A2MG, and MMP9, to promote Schwann cells (SCs) survival through Akt and ERK1/2 signal pathways [68,69,70]. The binding of tPA and MMP-9 to LRP1 induced c-Jun phosphorylation and ERK1/2 activation, with the response being inhibited by the LRP1 antagonist RAP, both in cultured rat and human SCs [71]. Intriguingly, c-Jun phosphorylation is a central event in the activation of the SCs repair program, supporting a model whereby LRP1 may also serve as an injury detection receptor in the peripheral nerve system [71]. The silencing of LRP1 with small interfering siRNA decreased phosphorylated Akt and increased activated caspase-3 in SCs, and comparable changes in the cell signaling were observed in LRP1 deficient murine embryonic fibroblasts [68]. Likewise, SC-specific LRP1 deletion exacerbated nerve injury, confirming the pro-survival effect of LRP1 signaling [72].

In macrophages, LRP1 deficiency increased apoptotic cell death and inflammation by impairing Akt activation [73]. LRP1 mediated tPA-induced M1 macrophage survival through a pathway involving ERK1/2, p90RSK, and p38 MAPK [74]. The LRP1 inhibition abrogated the lactoferrin-induced ERK1/2 signaling in primary rat osteoblastic cells [75]. Furthermore, the PDGF-mediated phosphorylation also resulted in an increased association of the adaptor protein Shc with LRP1, and the increased c-Jun N-terminal kinase (JNK) association at the plasma membrane prevented JNK translocation to the nucleus and gene regulation, thus inhibiting JNK signaling [76]. The JNK pathway, one of the major signaling cassettes of the MAPK signaling pathway, promotes apoptosis [81].

Thus, LRP1 appears to serve as a common receptor of multiple ligands to mediate their cytoprotective effects by activating different pro-survival signaling cascades. However, the specific contribution of LRP1 in the regulation of other cell death processes, such as necrosis and pyroptosis, has not been studied, and, therefore, whether LRP1 signaling also affects these phenomena is currently unknown.

### 4.2. Role of LRP1 in Inflammatory Signaling

LRP1 signaling also modulates the inflammatory response. LRP1 ligands increase in injured tissues and accelerate the resolution of the inflammatory response [46]. In macrophages, LRP1 was reported to play an essential role in mediating tPA anti-inflammatory cell signaling and the down-regulation of the expression of several cytokines [82]. Other LRP1 ligands, such as A2MG and MMP9, have a similar anti-inflammatory activity [47,69]. After ligand binding, the cytoplasmic fragment of LRP1 undergoes proteolysis by γ-secretases [83] and migrates to the nucleus, where it binds to the interferon regulatory factor-3 (IRF-3) and promotes its nuclear export and proteasomal degradation, thus limiting the expression of the pro-inflammatory genes in cultured fibroblasts and macrophages [84]. The LRP1-agonist complex was also found to inhibit the interleukin-1 receptor associated kinase-1 (IRAK-1), leading to the down-regulation of the NF-kB pro-inflammatory signaling pathway in vascular smooth muscle cells (VSMCs) [85].

LRP1 regulates the signaling of various membrane proteins, including the cell surface calreticulin (CRT) and the transforming growth factor-β (TGF-β) receptor. LRP1 acts as a signaling co-receptor for CRT and mediates the effects of the matricellular soluble protein thrombospondin 1 (TSP1), which regulates cell adhesion and migration [86]. LRP1 signaling also appeared to facilitate TGF-β activity and promoted fibroblast expansion and maturation, leading to increased collagen deposition and scar, whereas the disruption of the LRP1 signaling appeared to impair this same process. This experimental evidence may be independent of LRP1 signaling, and may rather be through a scaffold function of LRP1 on the cell membrane [87].

Altogether, these experimental evidences allow one to speculate that LRP1 represents a key mechanism inhibiting cell death, and promoting the resolution of the inflammation and the formation of a scar following tissue injury [88]. These functions make LRP1 a molecular target of interest in the setting of acute myocardial ischemia and infarction.

### 4.3. Role of LRP1 in Acute Myocardial Ischemia and Infarction

**Figure 1:**
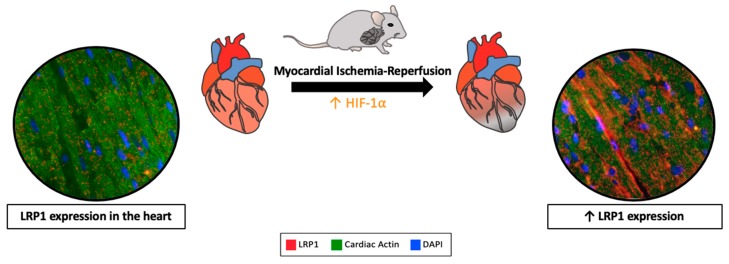
Lipoprotein receptor-related protein 1 (LRP1) expression is increased in the myocardial tissue after ischemia-reperfusion. Hypoxia upregulates LRP1 expression through induction of the hypoxia-inducible factor-1α (HIF-1α) in cardiomyocytes.

LRP1 expression is markedly enhanced during hypoxemia and/or ischemia [51,89,90] under the regulation of the hypoxia-inducible factor-1α (HIF-1α) in cardiomyocytes, as well as in cardiac fibroblasts [89,90,91,92] (Figure 1).

LRP1 was also strongly up-regulated in the myocardial tissue in the animal models of ischemia-reperfusion [51,90,91], as well as in patients with ischemic cardiomyopathy, supporting a role for LRP1 in IRI [51]. However, the specific role of LRP1 as a scavenger/signaling/scaffold receptor in cardiomyocytes has not been investigated until recently [51,90,92,93].

Multiple gain- and loss-of-function studies indicated that LRP1 protects against cardiomyocyte death and dysfunction through its ability to promote Akt- and ERK1/2-dependent survival pathways [51,77,78] (Table 2), consistent with what is described in neurons, Schwann cells, macrophages, fibroblasts, and osteoblasts [52,53,54,55,56,57,58,59,60,61,62,63,64,65,66,67,68,69,79,80].

LRP1 initiates the PI3K-Akt and MEK1/2-ERK1/2 signaling cascades shortly after the incubation of rat ventricular cardiomyocytes with A2MG, and induces protein synthesis and cell growth [77]. Treatment with a LRP1 stimulating antibody either in the presence or absence of A2MG resulted in comparable effects, while co-incubation with receptor associated protein (RAP), which sequesters LRP1 preventing ligand binding, significantly counteracted this hypertrophic response [77]. The effects elicited by A2MG-LRP1 signaling were abolished by the pre-treatment of cardiomyocytes with the MEK inhibitor UO126, the PI3K inhibitors LY294002 and wortmannin, and rapamycin, which inhibits mTOR downstream of Akt [77].

Ohashi et al. have previously shown that adiponectin through LRP1 and CRT promotes the neovascularization of ischemic muscle through a cyclooxygenase 2(COX-2)-dependent mechanism in endothelial cells [100]. It was later shown that the LRP1/CRT co-receptor system stimulates Akt phosphorylation and inhibits doxorubicin-induced apoptosis, whereas siRNA or blocking antibodies against LRP1 diminished the stimulatory effects of adiponectin on Akt activation and cardiomyocyte survival [78]. Although the specific involvement of LRP1 in the different death mechanisms (i.e., necrosis, apoptosis, and pyroptosis) needs to be further investigated, altogether, these data show that LRP1 signaling results in a protective effect in cardiomyocytes.

LRP1 has also been reported to be involved in cardiomyocyte lipid metabolism and calcium handling under ischemic conditions [90,91,93] (Table 2). Hypoxia-induced LRP1 expression led to an increased LRP1 scavenging function associated with an increased very low-density lipoprotein-cholesteryl ester (VLDL-CE) internalization. On the other hand, the deletion of LRP1 through lentiviral-mediated interfering RNA reduced the hypoxia-induced VLDL-CE uptake in HL-1 cardiomyocytes [90]. CE accumulated in the infarct area and in the bordering myocardium together with LRP1 up-regulation in a porcine model of ischemia-reperfusion [91]. This was speculated to be an attempt to reduce the oxidation of extracellular lipids through LRP1’s scavenging function, and/or a source of energy for the ischemic heart. Other investigators have proposed that cardiomyocytes exposed to hypoxic conditions in vitro become dysfunctional because of excessive lipid endocytosis, although this remains controversial [93]. In 2015, the Llorente-Cortés group observed that hypoxia-induced LRP1 up-regulation was concomitantly associated with reduced phosphorylation of the Ca^2+−^ dependent non-receptor tyrosine kinase proline-rich tyrosine kinase 2 (Pyk2), which in turn overloads the nucleus with the NF-kB and HIF-1α transcription factors, repressing sarcoplasmic reticulum Ca^2+^ ATPase (SERCA2) transcription and activity in HL-1 cardiomyocytes [93]. The modulation of the LRP1/pPyk2/HIF-1α axis may be useful to mitigate hypoxia-induced SERCA2 depletion, one of the main alterations in contractile dysfunction after AMI [93]. Notably, despite its negative effects on SERCA2 levels under hypoxic conditions, the proposed role for HIF-1α is protective in acute cardiac ischemia [101]. This may be due to the fact that, while ischemia occurs rapidly, hypoxia develops over a longer period of time.

Furthermore, various studies have highlighted the role of LRP1 in cardiac remodeling after AMI [92,94]. Enhanced LRP1-mediated cardiomyocyte intracellular CE accumulation up-regulated cathepsin S (CatS), a cysteine protease with the ability to degrade extracellular matrix (ECM) components, and altered structural and physical characteristics of secreted tropoelastin (TE), one of the main components of ECM [94]. LRP1 expression and protein levels markedly increased in the cardiac fibroblasts of the peri-infarct area at 10 and 21 days following permanent coronary artery ligation. The ERK1/2 expression and phosphorylation were strongly up-regulated in the infarct areas at 1, 10, and 21 days, while the pPky2 expression peaked in the infarct and peri-infarct areas at 10 and 21 days after AMI. The LRP1/pPyk2 axis up-regulated MMP-9 in cardiac fibroblasts, suggesting that LRP1 modulates the fibrotic response that follows the ischemic injury [92]. Taken together, these data allow one to speculate that LRP1 mediates the distinct spatial and temporal activation of ERK1/2- and Pyk2-dependent pathways in cardiomyocytes and fibroblasts, and may explain the differential contribution of these two signaling pathways in the immediate and later phases after AMI [92].

### 4.4. Effects of LRP1 Agonists in Ischemia Reperfusion

**Figure 2:**
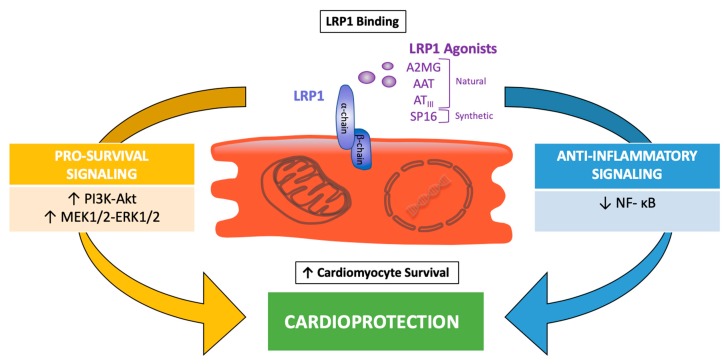
LRP1 protective signaling in myocardial ischemia reperfusion. Binding of natural serine proteinase inhibitors (SERPINs; *α*2-macroglobulin (A2MG), *α*1-antitrypsin (AAT), and antithrombin III (AT_III_)) and synthetic LRP1 agonists (SP16) induces cytoprotective signals in cardiomyocytes through the activation of pro-survival and anti-inflammatory signaling pathways.

The binding of SERPINs to LRP1 was shown to induce a pro-survival signal in several experimental settings, though its effects in a myocardial ischemia-reperfusion model remained unexplained until recently. LRP1 non-selective stimulation by SERPINs, such as with plasma-derived AAT, A2MG, and AT_III_, or with recombinant human AAT, significantly limited IRI [95,96,97,98,99]. Plasma derived AAT considerably inhibited cardiomyocyte death in vitro and in vivo [95], and the effects persisted when a clinically relevant scenario, such as prolonged ischemia (up to 75 min), delayed the administration of the drug (up to 30 min), and a large therapeutic index, was modeled in mice [96]. The use of different isoforms of recombinant fusion proteins composed of human AAT and Fc portion of IgGs, showed that the protective effects of AAT are independent from the inhibition of elastase activity [99]. Furthermore, it was also shown that treatment with LRP1 blocking antibody eliminated the benefits of AAT, suggesting that the cardioprotective effect of AAT is mediated by LRP1 signaling [51]. AT_III_ reduced the infarct size in mice, independent of its anticoagulant activity [98]. Plasma derived A2MG provided a similar cardioprotective signal across a large dose range in a mouse model of ischemia-reperfusion [97]. Collectively, these data demonstrate that distinct non-selective SERPINs that act as LRP1 agonists limited the IRI in mice (Figure 2). Based on these data, it has been recently proposed that selectively enhancing LRP1 signaling with a targeted agonist would have a comparable cardioprotective effect [51].

The short peptide LRP1 agonist, SP16, administered in mice intraperitoneally after reperfusion, significantly reduced the infarct size and preserved the left ventricular systolic function in a dose-dependent fashion [51]. The cardioprotective effects were maintained when SP16 was given subcutaneously at reperfusion or 30 minutes after reperfusion, whereas pre-treatment with the LRP1 blocking antibody abolished the benefits of SP16, confirming that a functional LRP1 receptor is indispensable to induce cardioprotection during experimental AMI [51]. Similarly, the inducible deletion of cardiac LRP1 using a Cre-lox system eliminates the infarct-sparing effects of SP16, confirming the specific role of cardiomyocyte LRP1 signaling in protecting the heart from acute ischemia [personal communication by the authors]. LRP1 stimulation through SP16 induced a rapid phosphorylation of the Akt protein kinase, associated with a reduction in proapoptotic to the antiapoptotic Bax/Bcl2 ratio and caspase-3 activity, in the heart tissue at 24 h from ischemia-reperfusion [51]. These findings support the concept that the upregulation of the RISK pathway mediates the protective effects of LRP1 in IRI (Figure 2).

Following IRI, the inflammatory response is necessary to promote infarct healing, but at the same time, the activation of key pro-inflammatory pathways induces a further loss of viable myocardium, leading to adverse ventricular remodeling and heart failure after AMI. Besides its pro-survival activity, LRP1 agonism has also proven to quench inflammatory signals. In vitro, the LRP1 agonist SP16 inhibited NLRP3 inflammasome activation and down-regulated NF-κB signaling following lipopolysaccharide (LPS) and Gp96 stimulation in a leukocyte assay, and treatment with the LRP1 blocking antibody dissipated these anti-inflammatory effects [51]. This is consistent with the significant reduction in infarct scar size and leukocyte infiltration in the peri-infarct border zone observed seven days from ischemia-reperfusion [51].

### 4.5. Clinical Experience with LRP1 Agonists in Ami

Promising pre-clinical studies with cyclosporine and other mitochondrial membrane permeability inhibitors failed to translate from bench to bedside, possibly as a result of inconsistent efficacy, dose- and time-dependent limitations, and concomitant standard-of-care treatment with P2Y_12_ receptor antagonists, which have been shown to independently provide, at least in part, some cardioprotective effects [19,102,103]. The data on LRP1 signaling and use of agonists show a much longer therapeutic window (up to two hours after reperfusion in the mouse), which would allow for treatment in time to provide a reduction in IRI.

Clinical data with plasma derived AAT, which functions as a non-selective LRP1 agonist, in patients with ST segment elevation acute myocardial infarction (STEMI), are limited to a pilot clinical feasibility trial (VCU-α1RT pilot study, clinicaltrials.gov, NCT01936896) [104]. A single intravenous administration of 60 mg/kg AAT within 12 hours from admission showed a favorable safety profile, and, when compared to a historical placebo-treated group from a parallel clinical trial, the plasma derived AAT was associated with a blunted acute inflammatory response as indicated by lower C reactive protein (CRP) levels [104] and reduced estimated infarct size, measured as the area under the curve for creatine kinase-myocardial band (CK-MB) [105]. There were no treatment-related serious adverse events. Of note, none of the patients treated with AAT had incident HF after one year, compared with one patient who died and nine patients who experienced heart failure (50%) in the placebo comparison group [105].

Selective LRP1 agonists are under clinical development. A phase I clinical trial testing with SP16 in healthy volunteers has been completed showing no toxicity(clinicaltrials.gov, NCT03651089) [106].

### 4.6. Controversies in the Field of Cardioprotection and Translational Outlook

The research field of cardioprotection has for decades been searching for a suitable strategy that could minimize, or prevent, lethal IRI after AMI. Despite many encouraging pre-clinical studies, which identified interventions that dramatically lower the infarct size, these studies have not translated into a clinically applicable drug or intervention in patients with AMI [2,5,7]. This gap between animal and clinical studies may have different explanations, including species differences; use of young and healthy animal models; poor experimental design; dose- and time-dependent limitations; and insufficient pre-clinical testing, including a lack of blinding by an independent validation by different laboratories [7,19,107]. For instance, the introduction of P2Y_12_ receptor antagonists as adjuvant antiplatelet therapy for all patients with AMI, has been proposed by some authors as a major factor for this lack of success in translating cardioprotective strategies into clinical settings [102,103]. P2Y_12_ receptor antagonists have potent anti-platelet effects and proposed cardioprotective effects, not attributable to the inhibition of platelet aggregation, through the modulation of signaling involving the RISK pathway [102,103]. Hence, it has been suggested that P2Y_12_ antagonists have already maximally triggered the RISK pathway, and other therapeutic interventions targeting the same pathway would give little additional benefit [102].

Future pre-clinical investigations would therefore need to further explore the cardioprotective molecular mechanisms of LRP1 modulation independently of the RISK pathway, including inducible cardiomyocyte-, endothelial-, leukocyte-, and fibroblast-restricted LRP1 deletion, in order to evaluate the cell-specific contribution of LRP1 stimulation during AMI, as well as testing in combination with a P2Y_12_ receptor antagonist in order to determine whether the new experimental approach would produce a synergic, more powerful, cardioprotective effect. It is worth noting, however, that in the pilot clinical trial with the LRP1 agonist, AAT, all of the patients had been pre-treated with a P2Y_12_ receptor antagonist, yet a benefit for the LRP1 agonist appeared to be maintained [104,105].

## 5. Conclusions

LRP1 is a multifunctional receptor that can be targeted following reperfusion to induce a cardioprotective signaling through the RISK pathway. The data from preclinical studies with non-selective and selective LRP1 agonists are promising, showing a large therapeutic window for intervention to reduce infarct size after ischemia reperfusion. A pilot clinical trial with plasma derived AAT, a naturally occurring LRP1 agonist, supports the potential translational value of LRP1 as a novel therapeutic target for cardioprotection. A phase I study with a selective LRP1 agonist has been completed showing no toxicity. These findings may open the way to early phase clinical studies with pharmacologic LRP1 activation in patients with AMI.

## Figures and Tables

**Table 1:** Lipoprotein receptor-related protein 1 (LRP1)-mediated pro-survival signaling pathways.

Signaling Pathway(s)	Ligand(s)	Co-Receptor(s)	Function	Ref.
↑ MAPK/ERK and PI3K-Akt, Src	PDGF	PDGFR-β	Regulation of cell migration, promotion of proliferation and survival	[59,60]
↑ ERK1/2, SFK, PI3K-Akt, PSD-95	tPA or α2M	Trk, NMDA	Promotion of cell survival	[61,62,63,64,65]
↑ ERK1/2, PI3K	MMP-9	-	Regulation of cell migration and promotion of cell survival	[66]
↑ PI3-Akt ↓ caspase-3, NF-kB, MAPKs	IVIg	-	Promotion of cell survival	[67]
↓ PI3-Akt ↑ caspase-3, NF-kB, MAPKs	RAP, LRP1-antibody, miR-205, siRNA	-	Inhibition of LRP1 promotes cell death	[51,77,78]

**Abbreviations**: MAPK—mitogen-activated protein kinases; SKF—Src family kinase; ERK—extracellular signal-regulated kinase; PI3K—phosphatidylinositol-4,5-bisphosphate 3-kinase; Akt—PI3K-protein kinase B; NF-kB—nuclear-factor kappa B; PDGF—platelet-derived growth factor; MMP-9—matrix metalloproteinase-9; IVIg—intravenous immunoglobulin; RAP—receptor associated protein. Upwards arrows (↑): upregulated signaling pathway(s); downwards arrows (↓): downregulated signaling pathway(s).

**Table 2:** LRP1-mediated signaling in acute myocardial ischemia and infarction.

Signaling Pathway(s)	LRP1 Ligand(s)	Effect/Function	Cell Types/Experimental Model	References
↑ PI3K-Atk and MEK1/2-ERK1/2	A2MG	↑ Protein synthesis and cell growth	Rat ventricular cardiomyocytes	[77]
↑ PI3k-Akt	Adiponectin	↓ Apoptosis and ↑ cell survival	Cardiomyocytes	[78]
↓ pPyk2/↑ HIF-1α/↓ SERCA2	VLDL, CE	Contractile dysfunction	Cardiomyocytes	[93]
↑ pPyk2/↑ MMP-9	-	Fibrosis	Cardiac fibroblasts	[92]
↑ Cathepsin secretion	CE	↑ CatS secretion and altered secreted TE (altered extracellular matrix)	Cardiomyocytes	[94]
↑ PI3K-Atk	Plasma derived AAT, Recombinant AAT, AT_III_, Plasma derived A2MG, SP16	↓ Apoptosis, ↓ infarct size, ↑ systolic function	Myocardial ischemia-reperfusion model	[51,95,96,97,98,99]

**Abbreviations**: CatS—cathepsin S; TE—tropoelastin; HIF-1*α—*hypoxia-inducible factor-1*α*; AAT—*α*1-antitrypsin; CE—cholesteryl ester; A2MG—*α*2-macroglobulin; VLDL—very low-density lipoprotein. Upwards arrows (↑): upregulated signaling pathway(s); downwards arrows (↓): downregulated signaling pathway(s).

## Abbreviations

AIF	Apoptosis-inducing factor
IL-1*β*	Interleukin-1*β*
IL-18	Interleukin-18
NLRP3	NACHT, LRR, and PYD domains-containing protein 3
DAMPs	Damage associated molecular patterns
PI3K	phosphatidylinositol-4,5-bisphosphate 3-kinase
Akt	PI3K-protein kinase B
MEK	mitogen-activated protein kinase kinase
ERK	Extracellular signal-regulated kinase
eNOS	Endothelial nitric oxide synthase
GPCR	G-protein-coupled receptors
PDK1	Pyruvate dehydrogenase kinase 1
mTORC	Mammalian target of rapamycin
TSC2	Tuberous sclerosis complex 2
ASC	Apoptosis-associated Speck-like protein containing a CARD
*α*2MR	*α*2-macroglobulin receptor
CD91	Cluster of differentiation 91
EGF	Epidermal growth factor
SEC	SERPIN-enzyme complex
SERPINs	Serine proteinase inhibitors
A2MG	*α*2-macroglobulin
AT_III_	Antithrombin III
tPA	Tissue-type plasminogen activator
PDGF	Platelet-derived growth factor
PDGFR-*β*	PDGF receptor-*β*
SFK	Src family kinase
NMDA	N-methyl-D-aspartate
PSD95	Postsynaptic density protein 95
MMP-9	Matrix metalloproteinase-9
RAP	Receptor associated protein
NF-kB	Nuclear-factor kappa B
MAPKs	Mitogen-activated protein kinases
IVIg	Intravenous immunoglobulin
MCAO	Middle cerebral artery occlusion
SCs	Schwann cells
siRNA	Short interference RNA
JNK	c-Jun N-terminal kinase
IRF-3	Interferon regulatory factor-3
IRAK-1	Interleukin-1 receptor associated kinase-1
VSMCs	Vascular smooth muscle cells
CRT	Calreticulin
TGF-*β*	Transforming growth factor-*β*
TSP1	Thrombospondin 1
HIF-1*α*	Hypoxia-inducible factor-1*α*
COX-2	Cyclooxygenase 2
VLDL	Very low-density lipoprotein
CE	Cholesteryl ester
Pyk2	Proline-rich tyrosine kinase 2
SERCA2	Sarcoplasmic reticulum Ca^2+^ ATPase
CatS	Cathepsin S
TE	Tropoelastin
LPS	Lipopolysaccharide
STEMI	ST segment elevation acute myocardial infarction
CRP	C reactive protein
IRI	Ischemia-reperfusion injury
LRP1	Low-density lipoprotein receptor-related protein 1
RISK	Reperfusion injury salvage kinase
AMI	Acute myocardial infarction
mPTP	Mitochondrial permeability transition pore
AAT	*α*1-antitrypsin
ROS	Reactive oxygen species
